# The Anti-Obesity Effects of Lemon Fermented Products in 3T3-L1 Preadipocytes and in a Rat Model with High-Calorie Diet-Induced Obesity

**DOI:** 10.3390/nu13082809

**Published:** 2021-08-16

**Authors:** Chih-Chung Wu, Yu-Wen Huang, Chih-Yao Hou, Ya-Ting Chen, Cheng-Di Dong, Chiu-Wen Chen, Reeta-Rani Singhania, Jie-Yin Leang, Shu-Ling Hsieh

**Affiliations:** 1 Department of Food and Nutrition, Providence University, Taichung 43301, Taiwan; wuccmail@gmail.com; 2 Department of Seafood Science, National Kaohsiung University of Science and Technology, Kaohsiung 81157, Taiwan; may2377234@gmail.com (Y.-W.H.); chihyaohou@gmail.com (C.-Y.H.); melodyyu.chen@gmail.com (Y.-T.C.); 3 Department of Marine Environmental Engineering, National Kaohsiung University of Science and Technology, Kaohsiung 81157, Taiwan; cddong@nkust.edu.tw (C.-D.D.); cwchen@nkust.edu.tw (C.-W.C.); reetasinghania@nkust.edu.tw (R.-R.S.); 4 Department of Food Science, National Pingtung University of Science and Technology, Pingtung 91201, Taiwan; dllm7117@yahoo.com.tw

**Keywords:** lemon fermented products, adipocytes, prevent obesity, 3T3-L1, lipid metabolism

## Abstract

Lemon (*Citrus limon*) has antioxidant, immunoregulatory, and blood lipid-lowering properties. This study aimed to determine the effect of the lemon fermented product (LFP) which is lemon fermented with *Lactobacillus* OPC1 to prevent obesity. The inhibition of lipid accumulation in 3T3-L1 adipocytes is examined using a Wistar rat model fed a high-fat diet to verify the anti-obesity efficacy and mechanism of LFP. Here, it was observed that LFP reduced cell proliferation and inhibited the lipid accumulation (8.3%) of 3T3-L1 adipocytes. Additionally, LFP reduced body weight (9.7%) and fat tissue weight (25.7%) of rats; reduced serum TG (17.0%), FFA (17.9%), glucose (29.3%) and ketone body (6.8%); and increased serum HDL-C (17.6%) and lipase activity (17.8%). LFP regulated the mRNA expression of genes related to lipid metabolism (PPARγ, C/EBPα, SREBP-1c, HSL, ATGL, FAS, and AMPK). Therefore, LFP reduces body weight and lipid accumulation by regulating the mRNA expression of genes related to lipid metabolism. Overall, our results implicate LFP as a potential dietary supplement for the prevention of obesity.

## 1. Introduction

Obesity has been linked to a number of chronic metabolic diseases, such as type 2 diabetes, hyperglycemia, dyslipidemia, and hypertension [1]. The World Health Organization (WHO) predicts that the number of obese adults will rise from 0.6 billion to 1.12 billion in the next ten years [2]. Thus, the prevention of obesity and its complications are very important. Obesity is caused by fat cell growth (adipogenesis) and increased cytoplasmic triglyceride accumulation (lipogenesis) [3]. Adipogenesis is the process of cell differentiation into adipocytes. The massive secretion of insulin will stimulate the performance of sterol regulatory element-binding protein-1c (SREBP-1c), peroxisome proliferators activated receptor γ (PPARγ) and CCAAT/enhancer-binding proteins α (C/EBPα) and promote the transformation of preadipocytes into mature adipocytes. Mature adipocytes secrete fatty acid synthesis (FAS) to synthesize triglycerides and accumulate them in the cells through fatty acid-binding protein 4 (FABP4) transportation [4]. In addition, adenosine monophosphate-activated protein kinase (AMPK) is thought to be a key marker in the therapy of metabolic diseases like obesity and type 2 diabetes [5]. Mature adipocytes also increase hormone-sensitive lipids by promoting the phosphorylation of AMPK. AMPK can inhibit triglyceride accumulation by reducing the secretion of adipose triglyceride lipase (ATGL) and hormone-sensitive lipase (HSL) [6].

Among citrus fruits, lemon (*Citrus limon*) production is estimated to be about 22 million tons annually in the world. Production of lemon continues to increase every year [7]. Lemon is rich in nutrients such as vitamin C, flavonoids, phenolic compounds, and citric acid [8]. Lemon possesses several beneficial properties like anti-oxidation, anti-cancer, immune function regulation, regulation of blood lipids and blood pressure, and the ability to promote wound healing [9,10,11]. Trovato et al. find that lyophilized citrus juices can lower triglyceride (TG) and levels of low-density lipoprotein-cholesterol (LDL-C) in rats while enhancing the levels of high-density lipoprotein-cholesterol (HDL-C) [12]. Therefore, *C. limon* juices are confirmed as a protection against hypercholesterolemia. Oboh et al. showed that lemon juice can reduce plasma total cholesterol (TC), TG, and LDL-C levels and increase HDL-C levels in a high-cholesterol diet which induces obesity in Wistar rats [13]. Hence, lemon has the capacity to benefit health effectively.

Fermentation by *Lactobacillus* can improve the spoilage of fruits and vegetables during long-distance transportation and extend the shelf life [14] and can increase the physiological functions of fruits or vegetables, such as improving anti-oxidant and anti-bacterial, anti-obesity, anti-cancer, and hypolipidemic ability [15,16,17,18]. The lemon that had been fermented proved to be functional. Previous studies show that anti-bacterial activity against *Salmonella typhimurium* and *Escherichia coli* O157: H7 was observed in *Lactobacillus plantarum* LS5 fermented sweet lemon juice [19]. Fermented lemon juice (FLJ) can prevent hepatic injury by lowering plasma aspartate aminotransferase activity (AST) and alanine transaminase activity (ALT) levels, hepatic lipid peroxidation, splenomegaly, and liver water in rats with carbon tetrachloride (CCL_4_)-induced liver injury [20]. Our previous study has shown that lemon fermented with *Lactobacillus* OPC1 into lemon fermented products (LFP) exhibited the activity of anti-oxidant enzymes, such as glutathione peroxidase (GPx), superoxide dismutase (SOD), catalase (CAT), and reduced the reactive oxygen species (ROS) content in Clone-9 cells. Moreover, it could maintain mitochondrial integrity and reduce oxidative stress damage by increasing the mitochondrial membrane potential [21]. In addition, LFP contains excellent physiological and bioactive compounds, such as polyphenols, vitamin C, flavonoid, and limonene [22]. Previous studies show that polyphenols, flavonoids, and limonene have anti-obesity effects [23,24]. However, there are no studies on the effects of lemon fermented products on the prevention of obesity.

In the present study, the efficacy and mechanism of LFP’s effects on lipid metabolism in 3T3-L1 preadipocytes and Wistar rats fed with high-calorie diets were investigated to establish its potent application in preventing obesity. Cell proliferation and lipid accumulation were considered indicators of the anti-obesity ability of LFP in cell models. Wistar rats were fed a high-calorie diet (HD) and administered LFP for 9 weeks, and the weight of the body, serum biochemical parameters, and mRNA expression of genes related to lipid metabolism (PPARγ, C/EBPα, SREBP-1c, HSL, ATGL, FAS, and AMPK) were then assessed.

## 2. Materials and Methods

### 2.1. Chemicals and Reagents

Dulbecco’s modified eagle high-glucose medium (DMEM), fetal bovine serum (FBS), penicillin/streptomycin, and trypsin were purchased from Gibco (New York, USA). Sodium bicarbonate, phosphate-buffered saline (PBS) were purchased from Uni-onward (Taipei, Taiwan). 3-isobutyl-methylxanthine (IBMX), dexamethasone (DEX), insulin, 3-(4,5-dimethyazol-2-yl)-2,5-diphenyltetrazolium bromide (MTT), Oil red O, formalin, triton X-100, TRIzol reagent, and chloroform were purchased from Sigma-Aldrich (St. Louis, USA). Isopropanol was purchased from J.T Baker (Pennsylvania, USA). The levels of total cholesterol (TC, CH202), TG (TR212), LDL-C (CH2657), HDL-C (CH2655), ketone body (RB1007), and lipase activity (LI118) commercial kits were purchased from RANDOX (Ireland, UK). The levels of glucose (ab65333), free fatty acid (FFA, ab65341), aspartate aminotransferase activity (AST, ab105135), alanine transaminase activity (ALT, ab105134), creatinine (ab65340), blood urea nitrogen (BUN, ab83362), sodium (Na, ab211096) and potassium (K, ab252904) commercial kits were purchased from Abcam (Cambridge, UK). Oligo dT, M-MLV Reverse transcriptase and RNase were purchased from Promega (Madison, USA). Primer and 2X SYBR Frist MM were purchased from Topgen (Kaohsiung, Taiwan).

### 2.2. Preparation of Lemon Fermented Products

Lemon and *Lactobacillus* OPC1 provided from Openmind Co. Ltd. (Kaohsiung, Taiwan). The citrus juicer machine squeezed the entire lemon into juice. Lemon juice with a Brix value of 7–10 °Brix and a pH of 2.5–2.8 was inoculated with 0.1% *Lactobacillus* OPC1 (powder, as determined by the weight of lemon juice) and incubated at 25 °C for 90 days of fermentation. Lemon fermented products (LFP) were obtained and sterilized by heating them at 80 °C for 1 min. LFP was prepared from the method developed by our previous study [21].

### 2.3. Cell Culture and Adipocyte Differentiation

**Figure 1:**
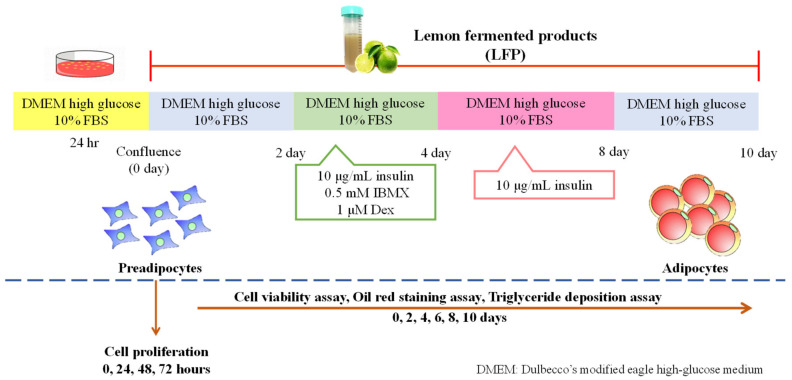
Experimental design of 3T3-L1 differentiation into adipocytes. FBS: fetal bovine serum; IBMX: 3-isobutyl-methylxanthine.

3T3-L1 preadipocytes obtained from the Bioresource Collection and Research Center (Hsinchu, Taiwan) were cultured in DMEM with 3.7 g/L sodium bicarbonate, 10% FBS, and 1% penicillin/streptomycin (100 U/mL) at 37 °C in a humidified atmosphere of 5% CO_2_. 3T3-L1 preadipocytes were incubated in 3 cm plates (5 × 10^4^ cells) for 24 h (0 day) and treated with different concentrations of LFP for 10 days. They were stimulated with an MDI mixture containing IBMX (0.5 Mm), insulin (10 μg/mL), and DEX (1 μM) on the second to the fourth day. Two days after stimulation with MDI (4th day), the cells were cultured in 10 μg/mL insulin medium on the fifth to the eighth day. Four days later (8th day), the cells were cultured in DMEM medium for two days. By the tenth day, full differentiation had been accomplished. (Figure 1). All results in the cell model were obtained from four independent experiments.

### 2.4. Cell Proliferation Assay

3T3-L1 preadipocytes were incubated in 3 cm plates (1 × 10^5^ cells) for 24 h and treated with 0.1, 0.5, 0.75, 1 mg/mL LFP for 72 h. The cell proliferation assay was conducted by withdrawing cultured cells followed by washing twice with 1mL PBS and incubated for 3 min at 37 °C and 5% CO_2_ after adding trypsin. The cells were then collected in DMEM medium and transferred to a 1.5 mL tube, where they were centrifuged for 3 min at 201× *g* and 25 °C. After that, the supernatant was extracted and combined with 4 °C PBS. 3T3-L1 preadipocytes were suspended in 20 μL of PBS with solution 13 (1 μL). (containing 286 μM DAPI and 80 μM acridine orange). NucleoCounter^®^ NC-3000™ fluorescence image cytometer (Chemometec, Allerod, Denmark) was employed for analysis via 8-chamber NC-Slides A8™.

### 2.5. Cell Viability Assay

The MTT reduction assay was used to determine cell viability [25]. 3T3-L1 preadipocytes were incubated in 3 cm plates (5 × 10^4^ cells) for 24 h. Cell treated by MDI combined 0.75 or 1 mg/mL LFP for 10 days. After 1 mL MTT (0.1 mg/mL) was applied, the optical density (OD) was measured at 570 nm. The cell viability (%) was expressed in terms of the percentage of viable cells in comparison to control.

### 2.6. Lipid Accumulation Assay

#### 2.6.1. Oil Red O Staining Assay

Oil Red O (in 60% isopropanol) was used to stain cells every two days during the differentiation process. The cells were gently washed with 1 mL PBS twice and 1 mL 10% formalin was added for 10 min. 1 mL Oil Red O solution was used for 30 min of staining. The plates were washed in water after the Oil Red O staining solution was removed. The stained lipid droplets were viewed on a microscope (Olympus, Tokyo, Japan). Then, 1 mL isopropanol was added and was shaken for 15 min. The optical density (OD) was measured at 510 nm. The quantitative oil red level (%) was expressed as the percentage of viable cells compared to control.

#### 2.6.2. Triglyceride Deposition Assay

The cells were washed twice with PBS to analyze the content of cellular triglycerides, then 70 μL of 0.5% Triton X-100 and scraped into 1.5 mL tube and mixed via a sonicator (Qsonica, CT, USA) for 30 s (power rating: 125 watts, frequency: 20 kHz, amplitude: 80%). The cell samples were assayed for TG content using assay kits and protein concentration was quantified by the method of Lowry [26]. The results were measured in mg of triglycerides per mg of cellular protein.

### 2.7. Animals and Treatments

Six-week-old Wistar rats (male, 250 ± 20 g) were purchased from BioLASCO Taiwan Co., Ltd. in Taipei. The Institutional Animal Care and Use Committees (IACUC) at the National Kaohsiung University of Science and Technology (Kaohsiung, Taiwan) approved all experimental protocols and animal care used in this research. The IACUC number is 0109-AAAP-014. Rats were kept in a cage under a 12 h light/12 h dark cycle at a controlled room temperature of 22 °C. After acclimating to the facility for 1 week, the rats were randomly divided into five groups (*n* = 14 per experimental group, *n* = 70) and fed a normal diet (ND) (58.0% carbohydrate, 13.5% lipid, 28.5% protein, 3.36 kcal/g), a high-calorie diet (HD) (45.0% carbohydrate, 45.0% lipid, 10.0% protein, 8.48 kcal/g), or HD combined with the low doses of LFP (HD+LLFP; 0.58 g/kg), HD combined with the medium dose of LFP (HD+MLFP; 1.73 g/kg), and HD combined with the high dose of LFP (HD+HLFP; 2.89 g/kg) for 9 weeks. The LFP dose used in the present study was determined based on this study of cell model by using the blood and body weight of rats. HD is made from sugar and soybean oil. LFP was administered by oral gavage four times a week. The ND group and HD group also consumed PBS by oral gavage. The study included measurements of body weight and food intake twice a week. The feed conversion rate (FCR) was derived by dividing the total weight gain (g) by diet consumption (g). Body weight-related organ weight (%) is (organ weight/body weight) * 100. The rats were fasted for 12 h before being carbon dioxide euthanized at the completion of the procedure. The CO_2_-based euthanasia displacement rate was 20–30% of the chamber volume per minute. For subsequent analysis, using cardiac punctures, blood samples were taken. The livers and epididymal fat tissues were removed and preserved at −80 °C. The blood was incubated at room temperature for 30 min before being centrifuged for 10 min at 1100× *g* and 4 °C. The serum (upper fraction) was drawn and processed for lipid analysis. Based on animal experiment ethics (replacement, reduction, refinement), only 10 rats were sacrificed in each group. In the part of animal growth characteristics and serum biochemical indicators, data analysis was carried out with 10 rats per group.

### 2.8. Serum Biochemical Parameters Determination Assays

Commercial kits were used to test the levels of glucose, TG, LDL-C, TC, FFA, HDL-C, lipase activity, ketone bodies, AST, ALT, creatinine, BUN, Na, and K according to the manufacturer’s prescribed protocols.

### 2.9. Histological Analysis

Liver and epididymis fat tissues were fixed with formalin (10%) and embedded in paraffin. The paraffin-embedded tissues’ parts (8 mm thick) were stained with haematoxylin and eosin (H&E) according to the Harris haematoxylin and eosin staining procedure [27]. The stained slides were observed with a microscope (Olympus, Tokyo, Japan). The area of adipocytes was analyzed by the Image-J software (U. S. National Institutes of Health, Bethesda, MD, USA). All results were obtained from five independent experiments.

### 2.10. Real-Time PCR

Total RNA was extracted using the TRIzol reagent, as directed by the manufacturer. Quantification of total RNA was conducted with an Epoch microvolume spectrophotometer system (Bio-Tek, Winooski, VT, USA). First-strand complementary (c) DNA synthesis by reverse-transcription (RT) was accomplished using RNase and M-MLV reverse transcriptase to transcribe poly (A)+ RNA with oligo-dT as the primer. Quantitative PCR analysis was carried out using SYBR green. Relative gene expression was quantified using a real-time PCR technique (LightCycler^®^ 96 Real-Time PCR System, Roche Life Science, Basel, Switzerland). Initial denaturation at 95 °C for 120 s was followed by 40 cycles at 95 °C for 5 s and 60 °C for 30 s for all assays. Denaturing at 95 °C for 10 s, cooling to 65 °C for 60 s, and then heated to 97 °C for 1 s in the melting analysis. Each cDNA was amplified using specific primers. The threshold cycle (Ct) values of target genes were normalized to the Ct values of β-actin. We used the comparative Ct method to calculate the fold changes in gene expression. The gene-specific primers used were β-actin as the control group, and the target genes were PPARγ, C/EBPα, SREBP-1c, HSL, ATGL, FAS, and AMPK (Table S1). In the experiment of genes related to lipid metabolism, each group was randomly selected (*n* = 5) for the independent experiment.

### 2.11. Statistical Analysis

The statistical analysis software SPSS for Windows, version 20.0, was used to examine the data (SPSS, Inc., Chicago, IL, USA). To determine the significance of discrepancies between two mean values, a one-way analysis of variance and Duncan’s test were used. A *p*-value of <0.05 was considered to indicate a statistically significant result. The different letters indicate that a group is significantly different from each other group (*p* < 0.05).

## 3. Results

### 3.1. Effect of LFP on Cell Proliferation in 3T3-L1 Preadipocytes

**Figure 2:**
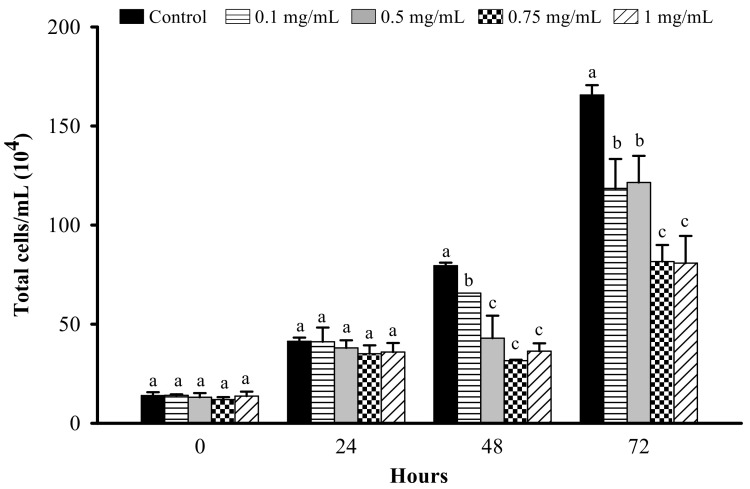
Effect of LFP on cell proliferation in 3T3-L1 preadipocytes. 3T3-L1 preadipocytes were treated or non-treated with 0.1, 0.5, 0.75, 1 mg/mL LFP for 72 h. The significance of difference in cell proliferation was evaluated by Duncan’s test. Data are the means ± SD (*n* = 4). Different letters (a–c) indicate significant differences among the different concentrations of the LFP group (*p* < 0.05).

The LFP at 0.1, 0.5, 0.75, and 1 mg/mL significantly reduced total cell number after 48 or 72 h (Figure 2). The reduction in cell proliferation is significantly better in cells treated with 0.75 and 1 mg/mL as compared to 0.1 and 0.5 mg/mL LFP at 48 and 72 h. Therefore, we would confirm the cell viability and lipid accumulation of 3T3-L1 adipocytes treated by MDI combined 0.75 or 1 mg/mL LFP.

### 3.2. Effect of LFP on Cell Viability in 3T3-L1 Adipocytes

**Figure 3:**
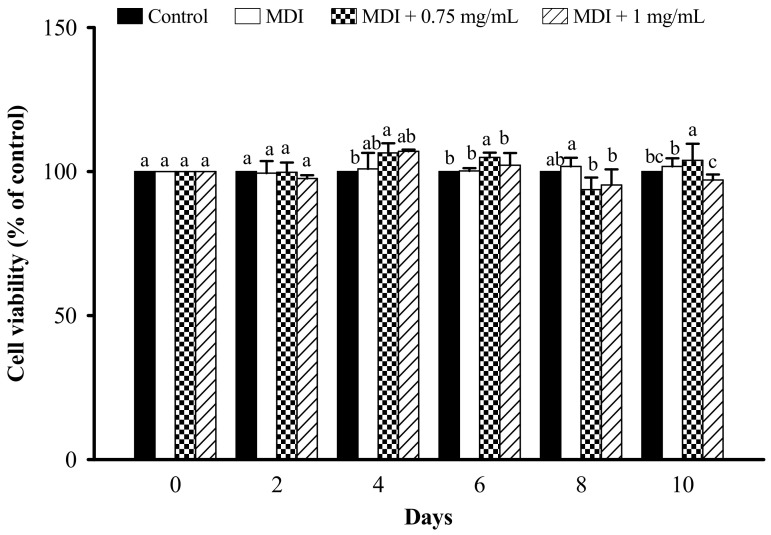
Effect of LFP on cell viability in 3T3-L1 adipocytes. 3T3-L1 adipocytes were treated or non-treated with 0.75, 1 mg/mL LFP for 10 days. Cells were collecting every two days, and non-differentiation preadipocyte is the control. The significance of difference in cell viability was evaluated by Duncan’s test. Data are the means ± SD (*n* = 4). Different letters (a–c) indicate significant differences among the different concentrations of the LFP group (*p* < 0.05).

Cell viability was measured by the MTT assay. Cells were incubated for 0–10 days by treatment with LFP at 0.75 and 1 mg/mL. The results showed that the cell viability of 3T3-L1 adipocytes cannot be reduced at 0.75 and 1 mg/mL LFP for 0–10 days (Figure 3).

### 3.3. Effect of LFP on Lipid Accumulation in 3T3-L1 Adipocytes

**Figure 4:**
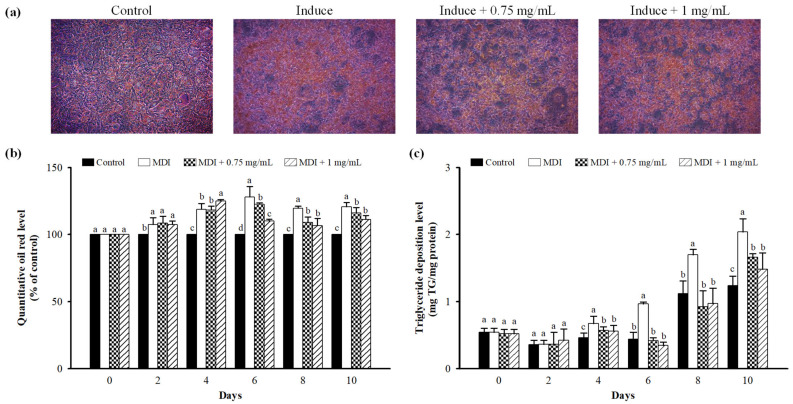
Effect of LFP on lipid accumulation in 3T3-L1 adipocytes. 3T3-L1 adipocytes were incubated in MDI medium containing 0.75, 1 mg/mL LFP for 10 days. Cells were collecting every two days. (**a**) Cell lipid accumulation levels were measured by staining with Oil Red O and observed under the microscope (×200)—picture only shows the final day. (**b**) 3T3-L1 adipocytes relative amount of control lipid accumulation (%). (**c**) Calculation followed by TG concentration (mg TG/mg protein) = mg TG/mg protein. Control: undifferentiated cells. The significance of difference in lipid accumulation evaluated by Duncan’s test. Data are the means ± SD (*n* = 4). Different letters (a–c) indicate significant differences among the different concentrations of the LFP group (*p* < 0.05).

Intracellular lipid content was determined using Oil Red O staining and TG analysis. Cells were incubated for 0–10 days with 0.75 and 1 mg/mL LFP. A change in adipocyte differentiation was observed at 10 days. LFP at 0.75 and 1 mg/mL significantly reduced intracellular lipid content on the 6, 8, and 10th day (*p* < 0.05). LFP at 0.75 and 1 mg/mL significantly reduced intracellular TG content on the 4, 6, 8, and 10th day (Figure 4).

### 3.4. Effect of LFP on Animal Characteristics in Rats Fed a High-Calorie Diet

The changes in animal characteristics of rats fed a high-calorie diet with LFP are shown in Table 1. After 9 weeks of feeding, the bodyweight of the HD group was significantly higher than the other groups. Rats treated with LLFP, MLFP, or HLFP exhibited a significant decrease in body weight and weight gain compared with the HD group (*p* < 0.05). However, no changes in the food intake and FCR were found among the different groups. This finding also shows that the HD caused a significant increase in fat tissue weight. Rats treated with LLFP, MLFP, and HLFP exhibited a significant decrease in fat tissue compared with the HD group. However, no changes in the heart, liver, spleen, and kidney weight were found among the different groups (Table S2).

### 3.5. Effect of LFP on Serum Biochemical Parameters in Rats Fed a High-Calorie Diet

The changes in serum biochemical parameters of rats fed a high-calorie diet with LFP are shown in Table 2. The levels of serum biochemical parameters were assayed. The serum TG, FFA, glucose, and ketone body levels of the HD rats were significantly higher than the ND group at the end of the 9-week feeding period. The serum HDL-C and lipase levels of the rats in the HD group were significantly lower than the ND group. LLFP, MLFP, and HLFP significantly reduced the serum TG and glucose levels compared with those obtained in the HD group (*p* < 0.05). MLFP and HLFP significantly reduced the serum FFA and ketone body levels and increased the HDL-C level and lipase activity compared with those obtained in the HD group (*p* < 0.05). The level of LDL-C, AST, ALT, creatinine, BUN, Na, and K from all groups show no differences.

### 3.6. Effects of LFP on Histological in Epididymal Adipose Tissue and Liver of Rats Fed a High-Calorie Diet

**Figure 5:**
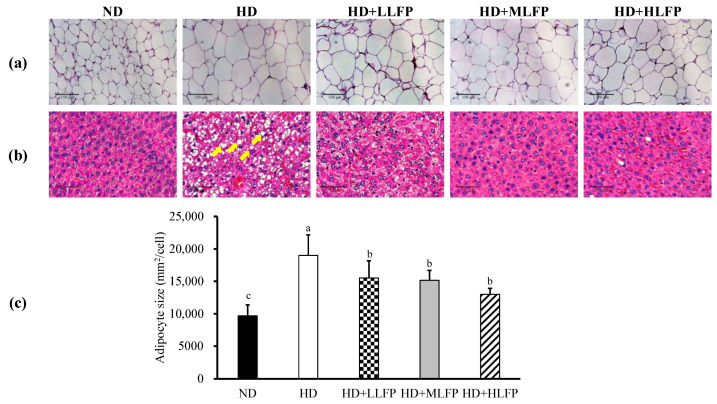
Effects of LFP on histological in epididymal adipose tissue and liver of rats a fed high-calorie diet. (**a**) Epididymal adipose tissues, (**b**) liver, and (**c**) adipocyte size. Epididymal adipose tissues and liver were stained with hematoxylin and eosin and were viewed under a microscope (×200). The significance of difference in adipocyte size was evaluated by Duncan’s test. Data are the means ± SD (*n* = 5). Different letters (a–c) indicate that the adipocyte size was statistically different from each other (*p* < 0.05). The groups are abbreviated as: Normal diet (ND), high calorie diet (HD), HD with low LFP (HD+LLFP), HD with medium LFP (HD+MLFP), HD with high LFP (HD+HLFP).

The sections of the epididymal adipose tissue and liver were stained using the H&E stain to be observed (Figure 5). LLFP, MLFP, and HLFP treatment significantly decreased the size of the adipose cells (Figure 5a,c). In addition, HD showed a notable increase in fat vacuoles (yellow arrow) when compared with ND. LLFP, MLFP, and HLFP showed dramatic decreases in fat vacuoles (both size and number) relative to HD (Figure 5b).

### 3.7. Effect of LFP on mRNA Expression of Genes Related to Lipid Metabolism in Epididymal Adipose Tissue of Rats a Fed High-Calorie Diet

**Figure 6:**
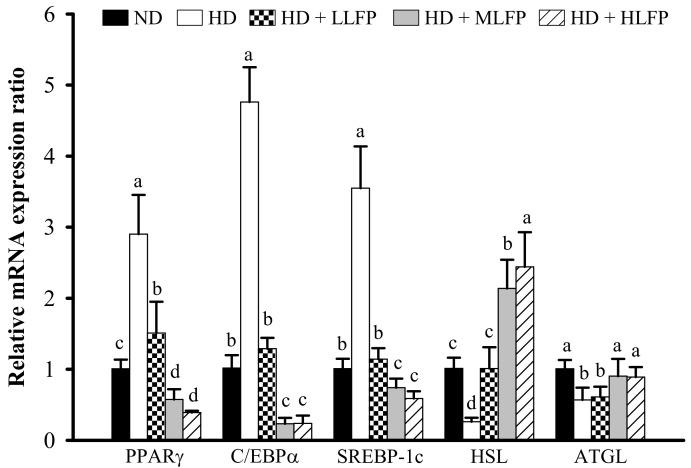
Effect of LFP on mRNA expression of genes related to lipid metabolism in epididymal adipose tissue of rats fed a high-calorie diet. The mRNA expressions of PPARγ, C/EBPα, SREBP-1c, HSL, and ATGL were determined by quantitative real-time PCR. The significance of difference in mRNA expression of genes related to lipid metabolism was evaluated by Duncan’s test. Data are the means ± SD (*n* = 5). Different letters (a–d) indicate that the mRNA expression was statistically different from each other (*p* < 0.05). The groups are abbreviated as: Normal diet (ND), high calorie diet (HD), HD with low LFP (HD+LLFP), HD with medium LFP (HD+MLFP), HD with high LFP (HD+HLFP).

To further explore the mechanism of LFP on lipid metabolism of rats fed a high-calorie diet, the mRNA expressions of genes related to lipid metabolism in epididymal adipose tissue were examined by real-time RCR. The mRNA levels of PPARγ, C/EBPα, and SREBP-1c in the HD group were significantly higher than in the ND group (*p* < 0.05). The LLFP, MLFP, and HLFP treated groups exhibited significantly decreased mRNA levels of PPARγ, C/EBPα, and SREBP-1c in epididymal adipose tissue compared with the HD group (*p* < 0.05). The mRNA levels of ATGL and HSL in the HD group were significantly lower than in the ND group (*p* < 0.05). The LLFP, MLFP, and HLFP treated groups exhibited significantly increased mRNA levels of HSL in epididymal adipose tissue compared with the HD group (*p* < 0.05), the MLFP and HLFP treated groups also exhibited significantly increased mRNA levels of ATGL in epididymal adipose tissue compared with the HD group (*p* < 0.05) (Figure 6).

### 3.8. Effect of LFP on mRNA Expression of Genes Related to Lipid Metabolism in Liver of Rats Fed a High-Calorie Diet

**Figure 7:**
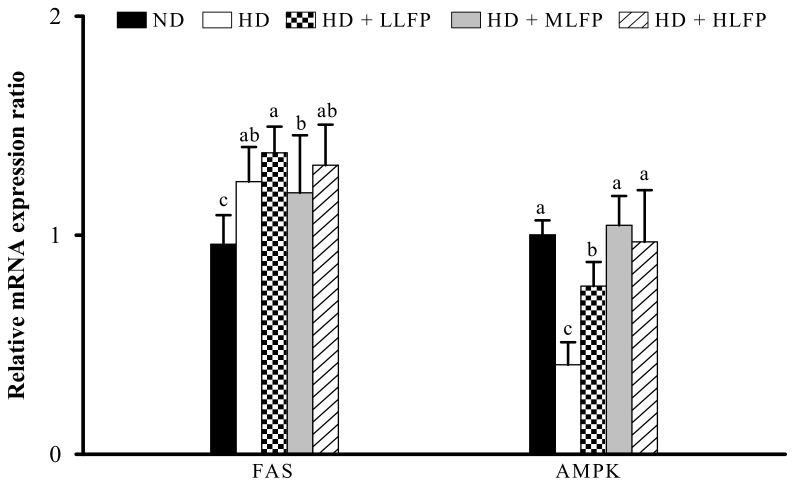
Effect of LFP on mRNA expression of genes related to lipid metabolism in the liver of rats fed a high-calorie diet. The mRNA expressions of FAS and AMPK were measured by quantitative real-time PCR. The significance of difference in mRNA expression of genes related to lipid metabolism was evaluated by Duncan’s test. Data are the means ± SD (*n* = 5). Different letters (a–c) indicate that the mRNA expression was statistically different from each other (*p* < 0.05). The groups are abbreviated as: Normal diet (ND), high calorie diet (HD), HD with low LFP (HD+LLFP), HD with medium LFP (HD+MLFP), HD with high LFP (HD+HLFP).

In addition, the mRNA expressions of genes related to lipid metabolism in the liver were examined. The mRNA levels of FAS in the HD group were significantly higher than in the ND group (*p* < 0.05). The LLFP, MLFP, and HLFP did not affect the mRNA levels of FAS in the liver of rats fed a high-calorie diet. The mRNA levels of AMPK in the HD group were significantly lower than in the ND group (*p* < 0.05). The LLFP, MLFP, and HLFP treated groups exhibited significantly increased mRNA levels of AMPK in the liver compared with the HD group (*p* < 0.05) (Figure 7).

## 4. Discussion

The differentiation and lipid buildup of adipocytes are linked to the development of obesity. Obesity can be prevented by lowering the number of adipocytes and lowering their lipid content [28]. In our study, we found LFP inhibited cell proliferation and reduced the lipid accumulation of 3T3-L1 adipocytes. Previous studies show that fermented soybean hull, *Monascus pilosus*-fermented black soybean extracts, and *Lactobacillus paracasei* subsp. *paracasei* NTU101-fermented tea can reduce the proliferation of 3T3-L1 cells and inhibit lipid accumulation in the 3T3-L1 cell [29,30,31]. These results show that fermented plants administration can reduce the proliferation of 3T3-L1 cells and inhibit lipid accumulation. In addition, our study showed that LFP can inhibit the cell proliferation of 3T3-L1 preadipocytes, but cannot reduce the cell viability of 3T3-L1 adipocytes. Wang et al. treated the 3T3-L1 cell with riceberry rice extract also found the same results [31].

The regulation of blood sugar, triglyceride, and low- or high-density lipoprotein cholesterol is key to obesity [32]. The findings of this study demonstrate that LFP can reduce serum glucose, lipid levels and regulate the electrolyte balance. According to previous studies, fermented plants can lower glucose and cholesterol levels in serum [16,33]. Different transcription factors are used to initiate preadipocyte differentiation and impact adipogenesis [34,35]. The master regulator of adipogenesis is thought to be PPARγ. Other adipogenic transcription factors, such as C/EBPα and SREBP-1c, are also involved in adipogenesis [36]. Hormones including cortisol and insulin stimulate them, which in turn activate PPARγ and C/EBPα. FABP4, FAS, HSL, and ATGL are all required for maintaining the phenotypic and function of adipocytes during the final phases of differentiation. [37]. In our study, we found that LFP can regulate the genes related to lipid metabolism (PPARγ, C/EBPα, SREBP-1c, ATGL, HSL, and AMPK). *Lactobacillus plantarum* EM fermented cabbage-apple (FCA) can reduce the mRNA expression of FAS, ACC, and SREBP-1c [38]. Fermented chestnut inner shell extract (FCCE) can reduce the mRNA or protein expression of FAS and SREBP-1c, and improve the protein expression of AMPK [39]. Multi-strain coupled fermented capsicum (EFC) can regulate the mRNA expression of PPARγ, C/EBPα, and HSL [40]. Ethanol extract from fermented *Curcuma longa* L. (FCE50) can reduce the mRNA expression of PPARγ, C/EBPα, FAS, and ACC, enhancing the mRNA expression of HSL and ATGL [41]. According to previous studies, fermented plants can reduce lipid accumulation by regulating the expression of lipid metabolism-related genes, especially PPARγ, C/EBPα, SREBP-1c, FAS, AMPK, ATGL, and HSL.

Fermentation can improve the nutrition and usefulness of food while also improving the biological activity of compounds [42]. Previous studies have shown that the fermentation of *Lactobacillus* and *Aspergillus* can increase the content of polyphenols, flavonoids, and antioxidant activity in fruits or vegetables [43]. Lemon juice fermented with *Lactobacillus plantarum* LS5 can increase its ascorbic acid and total phenol content, and increase anti-bacterial and anti-oxidant activities [19]. Citrus residues fermented with *Aspergillus* and found the content of naringenin and hesperidin can be increased after fermentation. And also found citrus residue has a good effect of inhibiting lipid accumulation of 3T3-L1 adipocytes after fermentation [44]. According to our previous research, the total polyphenol content in lemon juice is 4.14 ± 1.6 mg GAE, the vitamin C content is 23.0 ± 2.3 mg/mL, and the total flavonoid content is 53.5 ± 1.8 mg/L. After fermentation, total polyphenol content 25.8 ± 1.8 mg GAE, vitamin C content 55.2 ± 2.4 mg/mL, and total flavonoid content 75.2 ± 1.5 mg/L was present in LFP [21]. Total polyphenol, vitamin C, and total flavonoid content in lemon juice can be increased by 523, 140, and 40%, respectively, by fermentation. In addition, lemon also contains many aroma components, such as β-terpineol, o-octene, and limonene. In addition, lemon also contains many aroma components, such as β-terpineol, o-octene, and limonene.

In citrus essential oil, limonene is the most significant component [45]. Our previous study has found that the content of limonene in lemon juice is 6.13%. After fermentation, LFP has 10.55% limonene content [22]. Hence, limonene content can be increased by 4.42% after fermentation with *Lactobacillus*. Lone and Yun found that limonene can reduce the lipid accumulation of 3T3-L1 cells by increasing the mRNA or protein expression of HSL, FAS, and AMPK [46]. Jing et al. found that limonene can increase the serum HDL-C content, reduce the serum TG and the mRNA expression of SREBP-1c in mice. Santiago et al. found that supplementing D-limonene reduced lipid changes and lipid peroxide [47]. The above results indicate that limonene has the ability to regulate lipid metabolism. Hence, it can be speculated that the LFP has a good ability to prevent obesity because of the increase in the content of limonene through fermentation.

## 5. Conclusions

This study suggested that lemon fermented product (LFP) inhibited cell proliferation in 3T3-L1 preadipocytes and reduced lipid accumulation in cells. In addition, LFP can reduce the body weight of rats and lipid accumulation by regulating the mRNA expression of PPARγ, C/EBPα, SREBP-1c, ATGL, and HSL in adipose tissue, and the mRNA expression of AMPK in the liver. The LFP has the ability to regulate lipid metabolism, which is presumed to be caused by the increase in the content of limonene and total polyphenol after fermentation. These findings suggest a potential application for LFP in the prevention of obesity and related diseases.

## Figures and Tables

**Table 1:** Effect of LFP on animal characteristics in rats fed a high-calorie diet.

Characteristics	ND	HD	HD+LLFP	HD+MLFP	HD+HLFP
Body weight (g)	485.10 ± 7.72 ^b^	514.55 ± 15.10 ^a^	462.93 ± 13.13 ^b^	481.20 ± 13.74 ^b^	464.88 ± 14.53 ^b^
Weight gain (%)	113.76 ± 0.63 ^b^	134.63 ± 10.90 ^a^	109.95 ± 7.35 ^bc^	113.63 ± 4.79 ^b^	101.38 ± 5.56 ^c^
Food intake (g)	25.32 ± 0.87 ^a^	21.54 ± 0.32 ^a^	21.72 ± 0.71 ^a^	20.64 ± 0.84 ^a^	21.09 ± 1.30 ^a^
FCR (%)	5.63 ± 0.67 ^a^	6.81 ± 0.43 ^a^	6.27 ± 0.96 ^a^	6.48 ± 0.42 ^a^	7.11 ± 1.66 ^a^
Fat tissue (g)	1.46 ± 0.29 ^c^	2.80 ± 0.59 ^a^	2.14 ± 0.50 ^b^	2.29 ± 0.39 ^b^	2.08 ± 0.51 ^b^

Data are expressed as the means ± SD (*n* = 10). Different letters (a–c) indicate significant differences among each group (*p* < 0.05). Weight gain (%) is ((final weight-initial weight)/final weight) × 100. Feed conversion rate, FCR (%) is ((final weight-initial weight)/food intake) × 100. Fat tissue weight (%) is (Fat tissue weight/body weight) × 100. The groups are abbreviated as: Normal diet (ND), high calorie diet (HD), HD with low dose of LFP (HD+LLFP), HD with medium dose of LFP (HD+MLFP), and HD with high dose of LFP (HD+HLFP).

**Table 2:** Effect of LFP on serum biochemical parameters in rats fed a high-calorie diet.

Parameters	ND	HD	HD+LLFP	HD+MLFP	HD+HLFP
TG (mg/dL)	59.78 ± 7.72 ^c^	90.56 ± 13.88 ^a^	66.64 ± 8.72 ^bc^	68.14 ± 15.84 ^bc^	75.20 ± 9.01 ^b^
TC (mg/dL)	69.22 ± 0.63 ^a^	64.40 ± 7.20 ^a^	68.33 ± 6.75 ^a^	64.73 ± 8.64 ^a^	61.86 ± 9.23 ^a^
LDL-C (mg/dL)	6.44 ± 0.87 ^a^	7.88 ± 1.89 ^a^	7.11 ± 1.57 ^a^	7.88 ± 0.99 ^a^	7.43 ± 0.98 ^a^
HDL-C (mg/dL)	46.63 ± 0.67 ^a^	31.91 ± 4.78 ^c^	32.50 ± 4.44 ^c^	35.50 ± 3.60 ^b^	38.71 ± 5.85 ^b^
FFA (mmol/L)	1.11 ± 0.29 ^b^	1.45 ± 0.16 ^a^	1.33 ± 0.16 ^a^	1.18 ± 0.10 ^b^	1.19 ± 0.07 ^b^
Glucose (mg/dL)	52.00 ± 4.24 ^b^	78.57 ± 11.73 ^a^	56.63 ± 9.27 ^b^	58.50 ± 8.78 ^b^	55.57 ± 11.79 ^b^
Lipase (mmol/L)	144.78 ± 15.84 ^a^	116.64 ± 19.39 ^c^	122.06 ± 12.07 ^bc^	135.63 ± 11.40 ^ab^	141.89 ± 16.93 ^b^
Ketone body (mmol/L)	1.07 ± 0.03 ^c^	1.32 ± 0.09 ^a^	1.30 ± 0.08 ^ab^	1.24 ± 0.08 ^b^	1.23 ± 0.09 ^b^
AST (U/L)	167.30 ± 20.29 ^a^	163.25 ± 13.86 ^a^	164.50 ± 12.08 ^a^	164.67 ± 20.70 ^a^	166.57 ± 23.79 ^a^
ALT (U/L)	44.71 ± 5.71 ^a^	42.00 ± 5.59 ^a^	44.67 ± 5.28 ^a^	46.13 ± 7.02 ^a^	45.83 ± 8.70 ^a^
Creatinine (mg/dL)	0.63 ± 0.02 ^a^	0.64 ± 0.04 ^a^	0.63 ± 0.05 ^a^	0.61 ± 0.04 ^a^	0.59 ± 0.09 ^a^
BUN (mg/dL)	2.23 ± 0.63 ^a^	2.20 ± 0.68 ^a^	2.18 ± 0.59 ^a^	2.22 ± 0.41 ^a^	2.16 ± 0.73 ^a^
Na (mmol/L)	153.84 ± 1.44 ^a^	153.25 ± 1.14 ^a^	153.89 ± 0.95 ^a^	153.14 ± 1.60 ^a^	153.58 ± 1.08 ^a^
K (mmol/L)	7.34 ± 0.68 ^a^	7.11 ± 0.39 ^a^	6.75 ± 0.49 ^a^	6.50 ± 0.60 ^a^	6.26 ± 0.42 ^a^

Data are expressed as the means ± SD (*n* = 10). Different letters (a–c) indicate significant differences among each group (*p* < 0.05). The groups are abbreviated as: Normal diet (ND), high calorie diet (HD), HD with low dose of LFP (HD+LLFP), HD with medium dose of LFP (HD+MLFP), HD with high dose of LFP (HD+HLFP).

## Data Availability

Data are available from the corresponding author upon reasonable request.

## Supplementary Materials

The following are available online at https://www.mdpi.com/article/10.3390/nu13082809/s1, Table S1: Specific primer of real-time PCR analysis in this study. Table S2: Effect of LFP on body weight relative organ weight in rats fed a high-calorie diet.
