# Supplementary material for: The Anti-Obesity Effects of Lemon Fermented Products in 3T3-L1 Preadipocytes and in a Rat Model with High-Calorie Diet-Induced Obesity
Source: Nutrients. 2021 Aug 16;13(8):2809. doi: 10.3390/nu13082809 (PMC8398352; doi:10.3390/nu13082809)
Supplement: Supplementary file 1 [file nutrients-13-02809-s001.zip › Supplementary Table S1.(Specific primer).pdf]

Supplementary Table S1. Specific primer of real-time PCR analysis in this study.

Gene (rat)	Primer sequence (5′-3′)	Reference (NCBI GenBank)
β-actin	F- AGGCCCCTCTGAACCCTAAG<br>R- CAGCCTGGATGGCTACGTACA	NM031144.3
PPARγ	F- AAGTTTGAGTTTGCTGTGAAGTTC.A<br>R- CGATGGGCTTCACGTTCAG	NM001145366.1
C/EBPα	F- CATCGACTTCAGCGCCTACA<br>R- CCGCTTTGTGATTGCTGTTG	NM013154.2
SREBP-1c	F- CGGGACAGCTTAGCCTCTACA<br>R- CGGCCACAAGAAGTAGATCA	NM001276707.1
HSL	F-GTCACGCTACATAAAGGCTGCTT<br>R-CAGCCCGATGGAGAGAGTCT	NM012598.1
ATGL	F-GGCCACTGCCATGATGGTA<br>R-GCAGCCACTCCAACAAACG	NM001108509.2
FAS	F-TCTCCTGTTGACCCAGAGCAT<br>R-TGGGCCAGAATGGCATCT	NM017332.1
AMPK	F-GTGGATCGCCAAATTATGCA<br>R-AACCTCAGGACCCGCATACA	NM023991.1

F: Forward primer. R: Reverse primer.
