# Supplementary material for: The Anti-Obesity Effects of Lemon Fermented Products in 3T3-L1 Preadipocytes and in a Rat Model with High-Calorie Diet-Induced Obesity
Source: Nutrients. 2021 Aug 16;13(8):2809. doi: 10.3390/nu13082809 (PMC8398352; doi:10.3390/nu13082809)
Supplement: Supplementary file 1 [file nutrients-13-02809-s001.zip › Supplementary Table S2.(Related organ weight).pdf]

Supplementary Table S2. Effect of LFP on body weight relative organ weight in rats fed a high-calorie diet

**Organ**	**ND**	**HD**	**HD+LLFP**	**HD+MLFP**	**HD+HLFP**
Heart (g)	0.30 ± 0.02[a]	0.28 ± 0.02[a]	0.30 ± 0.02[a]	0.30 ± 0.03[a]	0.30 ± 0.03[a]
Liver (g)	3.19 ± 0.28[a]	3.17 ± 0.28[a]	3.17 ± 0.36[a]	3.14 ± 0.21[a]	3.09 ± 0.20[a]
Spleen(g)	0.20 ± 0.03[a]	0.17 ± 0.02[a]	0.17 ± 0.03[a]	0.18 ± 0.05[a]	0.17 ±0.01[a]
Spleen(g)	0.20 ± 0.03[a]	0.17 ± 0.02[a]	0.17 ± 0.03[a]	0.18 ± 0.05[a]	0.17 ±0.01[a]
Kidney(g)	0.80 ± 0.05[a]	0.74 ± 0.03[a]	0.73 ± 0.05[a]	0.73 ± 0.07[a]	0.73 ± 0.07[a]

Data are expressed as means ± SD (n=10). Different letters indicate significant differences among each group ($P<0.05$). Body weight related organ weight (%) is (organ weight/body weight)*100. The groups are abbreviated as: Normal diet (ND), High calories diet (HD), HD with low dose of LFP (HD+LLFP), HD with medium dose of LFP (HD+MLFP), HD with high dose of LFP (HD+HLFP).
